# Differential Phononic Crystal Sensor: Towards a Temperature Compensation Mechanism for Field Applications Development

**DOI:** 10.3390/s17091960

**Published:** 2017-08-25

**Authors:** Simón Villa-Arango, David Betancur Sánchez, Róbinson Torres, Panayiotis Kyriacou, Ralf Lucklum

**Affiliations:** 1 Biomedical Engineering Research Group (GIBEC), EIA University, Envigado 055428, Colombia; david.betancur8@eia.edu.co (D.B.S.); robinson.torres@eia.edu.co (R.T.); 2 Research Centre for Biomedical Engineering (RCBE), University of London, London EC1V 0HB, UK; p.kyriacou@city.ac.uk; 3 Institute for Micro and Sensor Systems (IMOS), Otto-von-Guericke University, Magdeburg 39106, Germany; ralf.lucklum@ovgu.de

**Keywords:** differential measurement, temperature compensation mechanism, phononic crystal, point of care test, transmission line model

## Abstract

Phononic crystals are resonant structures with great potential to be implemented in applications as liquid sensors. The use of the symmetry reduction technique allows introducing relevant transmission features inside bandgaps by creating defect modes in a periodic regular structure. These features can be used as measures to quantify changes in the speed of sound of liquid samples that could be related to the concentration of analytes or the presence of pathogens among other interesting applications. In order to be able to implement this new technology in more challenging applications, such as biomedical applications, it is necessary to have a very precise and accurate measurement. Changes in temperature greatly affect the speed of sound of the liquid samples, causing errors in the measurements. This article presents a phononic crystal sensor that, by introducing additional defect modes, can carry out differential measurements as a temperature compensation mechanism. Theoretical studies using the transmission line model and analytes at various temperatures show that the proposed temperature compensation mechanism enhances the performance of the sensor in a significant way. This temperature compensation strategy could also be implemented in crystals with different topologies.

## 1. Introduction

Phononic crystals (PnCs) are periodic composite materials with a spatial modulation of their acoustic properties. This feature allows PnC to selectively control the transmission of acoustic and elastic waves in liquids and solids respectively. One interesting feature of PnCs is the formation of bandgaps, which are frequency ranges over which acoustic and/or elastic mechanical waves are not transmitted. The most important properties to be taken into account when designing phononic crystals are geometry and the acoustic impedance of the constituent materials [1,2].

Due to the unique capability exhibited by phononic crystals to control the transmission of waves, scientists have presented a variety of interesting applications among which are: waveguides, acoustic super lenses, multiplexing/de-multiplexing systems, acoustic cloaking structures, and more recently, sensors [3,4,5].

PnC sensors can be designed using the symmetry reduction technique. This technique relies on the introduction of defects in an otherwise regular PnC structure to generate resonant modes inside bandgaps. These modes provide a unique sensor signal due to their resonance frequency being linked to the acoustic properties of liquid samples under test. Point, line, or plane defects can be used to generate relevant transmission features in PnC sensors. The material composing these defects is usually the analyte, thus, becoming also a constituent element of the phononic crystal structure. The resonance modes located inside the bandgap, also called defect modes, are usually found as maximums of transmission well elevated above ground noise and well separated from other transmission features like the borders of bandgaps, who help generating adequate boundary conditions for measuring the characteristics of these peaks [6].

It has been previously shown, both theoretically and experimentally, that the resonance frequency of the cavity mode generated by the disruption of the periodicity in one-dimensional and two-dimensional PnCs is linked to the relevant acoustic material properties of the analyte [7,8,9,10,11]. Recent experimental realizations showed that the sensitivity of the speed of sound of liquid mixtures is appropriate to analyze mixtures in real applications [12,13].

There are many groups currently working on PnC sensors and some of the most interesting applications of this new technology are the measurement of the concentration of analytes in complex mixtures or the conversion rate in micro-reactors. Other interesting applications are the performance of point of care testing due to the portability of the technology [5,6,7,8,9,10,11,14,15,16].

The simulation of resonant structures and especially of phononic crystals can be performed using diverse numerical methods like the finite difference time domain (FDTD), the plane wave expansion (PWE) method, and layer multi-scattering theory (LMST) among others [17,18,19].

Multi-layered phononic crystals, like the structure presented by Villa-Arango et al. [9], are crystals whose simulation can also be performed using simulation methods that use a lateral miniaturization of the structure, like the transmission line model, TLM [20]. This method has been widely used to simulate resonant structures and even phononic crystal sensors showing accurate results despite the use of a 1D model [5,21,22].

The TLM is based on an acoustic transmission line derived from an electrical transmission line by using an analogy between the electrical impedance and the acoustic impedance. The model simulates the transmission of longitudinal waves through semi-infinite media and calculates the transmission and reflection coefficients by using the cumulative effective acoustic impedance, $Z_{L}$. It is important to notice that the effective acoustic impedance, which depends on the frequency, *f*, and the dimensions of the layers, *e*, is different from the characteristic acoustic impedance of the material, $Z_{c}$, which is equal to the density, ρ, multiplied by the speed of sound of the material c. Equation (1) shows how the calculation of the effective acoustic impedance throughout each layer, i, of the crystal is performed. The concept considers the reflection and transmission of waves not only at the interfaces between layers, but also inside each one of them [20].

(1) $$Z_{L\left(i+1\right)}=Z_{c\left(i\right)}\frac{Z_{L\left(i\right)}+jZ_{c\left(i\right)}\tan\left(2\pi fe/c\right)}{Z_{c\left(i\right)}+jZ_{L\left(i\right)}\tan\left(2\pi fe/c\right)},$$

One of the great challenges when working with liquid samples is that their properties vary in a significant way when ambient conditions are not stable, especially with a fluctuating temperature. Bulky temperature control systems have been implemented as a solution, however, these systems cannot be used in applications where portability is mandatory, like in point of care testing or other field applications. Bilanuk et al. showed the effect that temperature, T, has in the speed of sound, c, of distilled water, Equation (2) [23,24].

(2) $$c\;=\;1.40238744\times 10^{3}\;+\;5.03836171T\;-\;5.81172916\times 10^{-2}T^{2}+3.34638117\times 10^{-4}T^{3}-\;1.48259672\times 10^{-6}T^{4}+3.16585020\times 10^{-9}T^{5},$$

Different alternatives to mitigate the effect of temperature without using bulky temperature control systems have been proposed. The use of differential measurements has been one of the most effective solutions. They use a second signal and a reference with known properties that allows compensation for the effect of temperature. The advantage of using these devices is that any external influence of pressure, or temperature, among others, will affect both the reference and the sample. Kaya et al. recently developed a theoretical study about an acoustic phononic crystal Mach–Zender interferometer with two signal paths, one for the reference and one for the sample and could be used as a temperature compensation mechanism [25].

This article presents a phononic crystal sensor with a temperature compensation mechanism that allows performing differential measurements. The PnC was designed using three defect modes instead of one like previous approaches. The differential PnC sensor presented in this article uses a single wave path for obtaining the information about the temperature and the analyte which facilitates the development and implementation of new sensors since all the information needed for characterising the liquid sample and compensating the temperature is contained in a single frequency spectra.

## 2. Materials and Methods 

**Figure 1:**
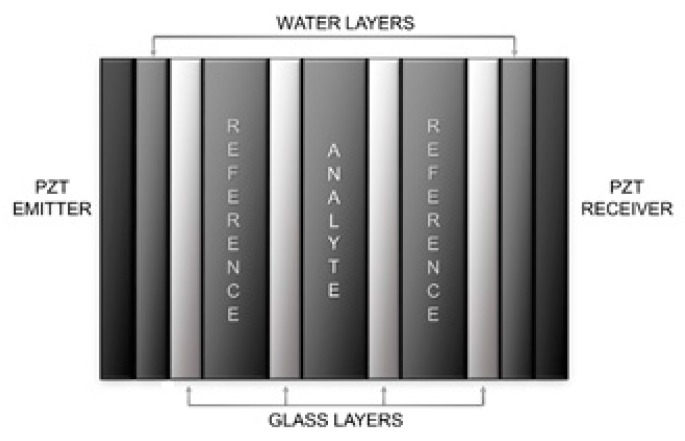
Differential phononic crystal sensor designed with three defect modes.

The PnC sensor presented on this work is comprised of a series of consecutive thin layers with different acoustic properties. The spatial modulation of the acoustic properties allows it to exhibit a wide bandgap. For the generation of the relevant characteristic features that are used in the measurement of the acoustic properties of the liquid samples the technique of symmetry reduction was used. A total of three defect modes were introduced into the crystal by altering the dimensions of the three central liquid layers. The designed structure can be observed in Figure 1. The central layer is composed by the analyte, while the two adjacent liquid layers are filled with a reference material. The dimensions of the reference and central layers were calculated using distilled water, therefore, making the three layers equal. The properties of the materials of each layer, as well as their dimensions, are shown in Table 1.

The differential PnC sensor was studied numerically using the transmission line model, TLM. This method was selected due to its performance on multi-layered structures. The TLM delivers the reflection and transmission coefficients of structures composed of thin isotropic layers. These coefficients are used to follow the frequency of characteristic transmission features generated by the technique of symmetry reduction on the frequency response of the sensor. To calculate the effective acoustic impedance of each layer, a program was coded in the mathematical software, MATLAB. The parameters used in the simulations are the properties of the layers (density, speed of sound, and layer thickness), the properties of the frequency sweep (initial frequency, steps, and step value) and finally the temperature at which the simulation is performed.

The speed of sound of the materials is closely related to the temperature, therefore, the value of the speed of sound of the layers composed of distilled water is adjusted using the equation described by Bilanuk et al. Equation (2).

**Figure 2:**
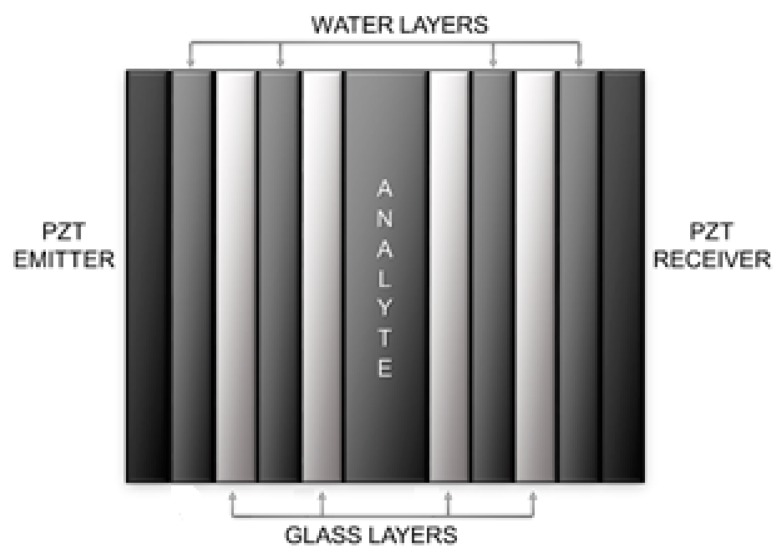
Control phononic crystal sensor designed with only one defect mode.

To be able to compare the results and observe the benefits obtained with the proposed differential PnC sensor, the simulation of an additional control sensor, Figure 2, was performed. The properties of the layers of the structure of the control PnC sensor are shown in Table 1. The control PnC sensor was designed using only one defect mode instead of three. 

A theoretical study was performed to evaluate the behaviour in frequency of the differential phononic crystal sensor designed. Each simulation was realised using the same parameters for the control PnC sensor and the differential PnC sensor. Distilled water was used as analyte and the response of both sensors was compared, paying special attention to the transmission maximums present in the centre of the bandgap, since these are the ones that carry the most relevant information about the changes in the speed of sound of the analyte. Temperature ranges going from 3 °C to 43 °C were used to evaluate the impact of temperature on the frequency spectrum of the structures. The formula of Bilanuk et al. Equation (2) was used to calculate the speed of sound of distilled water at the different temperatures of the study. Both crystals have a working frequency around 2 MHz, therefore, the initial frequency selected to perform the studies was 100 KHz, with steps of 1 Hz. Attenuation and losses were not included in the simulations because the aim of the study was to determine the impact of temperature on the frequency of relevant transmission features and how the temperature compensation mechanism could help improve the performance of the sensor. 

## 3. Results and Discussion

**Figure 3:**
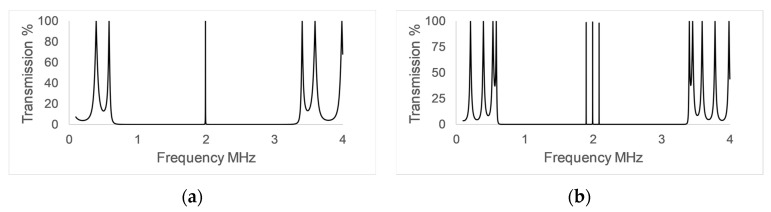
Simulation results using the TLM of the control PnC sensor (**a**) and the differential PnC sensor (**b**). Distilled water was used as analyte.

Figure 3 shows the frequency response of the control (a) and differential (b) PnC sensors at room temperature. The results of the computational simulation using the transmission line model show a well-defined bandgap on both sensors, ranging from 500 KHz to 3.5 MHz, resulting in a good bandwidth of 3MHz. The transmission spectrum of the control sensor structure has a maximum transmission located in the centre of the rejected band, while the frequency response of the differential sensor structure has three transmission maximums instead of one. The response presented by both sensors shows that when using the symmetry reduction technique, transmission maxima are generated within the rejected band, as already shown in previous studies [9], however, it is interesting to note that the use of three defect modes with the same characteristics generates three maxima in the center of the band.

The transmission maxima generated by the disruption in the symmetry of the PnC structure present a high-quality factor. This is mainly due to the effect exerted by the rejected band on them. Although the glass used in the solid layers of the PnC does not have an acoustic impedance as high as steel or other common solids, the impedance ratio between glass and distilled water is high enough to generate a bandgap with good depth and bandwidth.

The temperature compensation mechanism relies on the measurement of the frequency of the three transmission maxima observed at the centre of the bandgap of the differential sensor, Figure 3b. The design of these sensors is made so that only one of these three peaks is affected by the variations in the speed of sound of the analyte. This is achieved by introducing three defects and filling only one of them with the analyte, leaving the other two with a known material. In this case, distilled water. The peaks that are not sensitive to the changes in the analyte layer are used as a reference, thus, enabling the performance of differential measurements.

**Figure 4:**
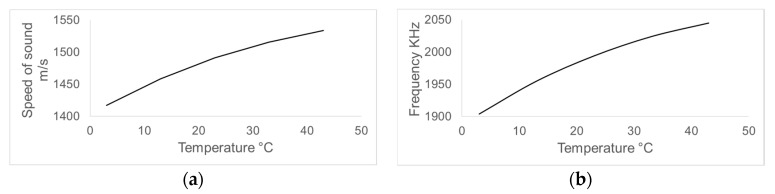
Behaviour of the speed of sound of distilled water (**a**) and the frequency of the central peak of the control phononic crystal (**b**) when temperature is varied from 3 °C to 43 °C.

Changes in temperature significantly affect the speed of sound of distilled water, causing the relevant frequency characteristics in PnC sensors to shift in frequency. Figure 4 shows how temperature variations ranging from 3 °C to 43 °C are related to the speed of sound of the analyte (a) and the frequency of the central maxima of transmission of the control PnC sensor (b).

By increasing the temperature at which the simulations are performed, the speed of sound in distilled water increases from 1416 m/s to 1533 m/s, which causes the relevant transmission maxima to move towards higher frequencies in the spectrum.

**Figure 5:**
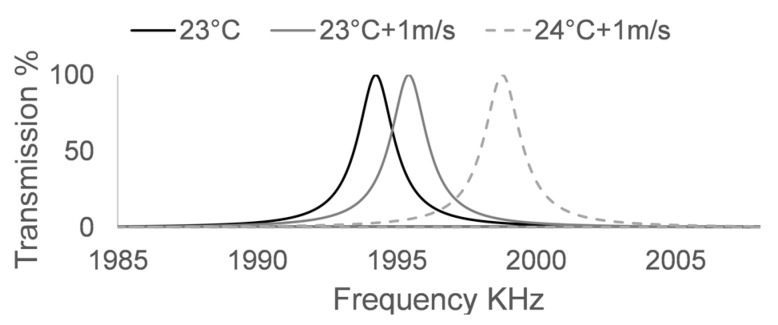
Effect of increasing 1 °C on simulations using the control PnC sensor. The black line shows the analyte at 23 °C, the grey line shows an increase of 1 m/s on the analyte and the dotted line shows the effect of adding 1 °C to the simulation.

Figure 5 shows the effect that a change of 1 °C would have in a test in which it is required to identify a variation of 1 m/s in a liquid sample using the control sensor shown in Figure 2. Increasing the speed of sound by 1 m/s makes the peak displace itself in frequency by 1.182 kHz. Further increasing the temperature by 1 °C makes it go to higher frequencies, displacing itself by another 3.353 KHz. The quality factor of the central transmission maxima is good, which makes it easy to identify and quantify small changes in frequency, however, a change of only 1 °C, generates great difficulties in identifying these changes, since its influence in the frequency position of the transmission maxima is almost three times greater than that of a change of 1 m/s.

**Figure 6:**
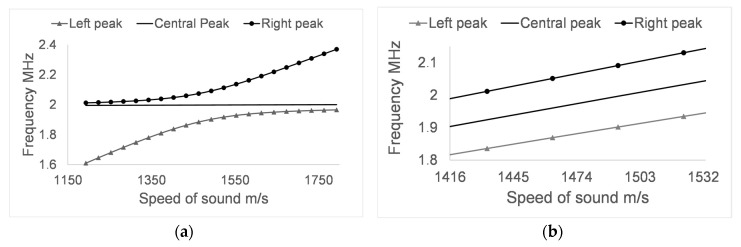
(**a**) Influence of changes in the speed of sound of the analyte layer (**a**) and the temperature (**b**) on the relevant transmission peaks of the differential PnC sensor.

After analysing the impact of varying the temperature in the control PnC sensor, the behaviour of the differential PnC sensor proposed in this work was analysed. In order to observe the performance of the temperature compensation mechanism in the differential sensor, two simulations were carried out. The first simulation was realised performing a variation of the speed of sound in the analyte layer, Figure 6a. The second simulation, Figure 6b, was performed by changing only the temperature, assuming that the analyte does not undergo further variations.

**Figure 7:**
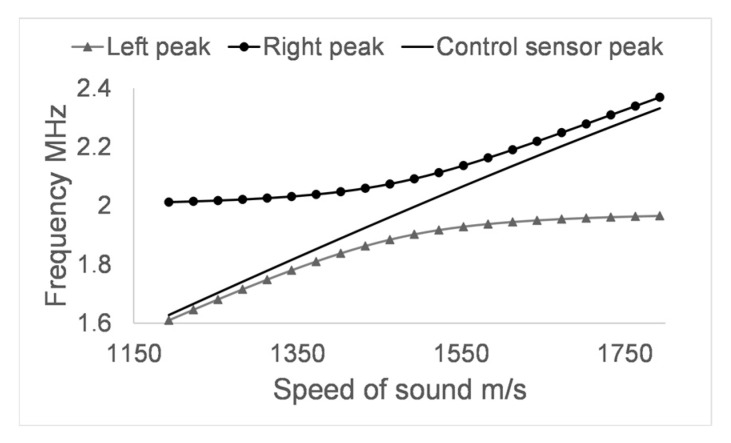
Relationship between the lateral peaks of the differential PnC sensor and the central peak of the control PnC sensor when the speed of sound of the analyte layer is varied.

The frequency response of the three central transmission maxima, Figure 6a, shows that the central maximum remains stable at 2 MHz when making changes in the speed of sound of the analyte. However, the other two transmission maxima suffer significant variations in frequency, which increase in magnitude as the lateral maxima move away from the central one. It is very interesting to observe how the behaviour of the two peaks that accompany the central peak is asymptotic with respect to the frequency response of the central peak of the control PnC sensor, Figure 7.

Figure 6b shows how, by varying the temperature from 3 °C to 43 °C, the frequency position of the transmission maxima was steadily increased, including the central transmission maximum. This behaviour allows the use of the central maximum, which is sensitive to changes in temperature, but not to changes in the properties of the analyte, as a reference to perform differential measurements and thus to have a temperature compensation mechanism.

It is important to note that although in Figure 6a the central maxima appear to be stable at 2 MHz over the entire range of sound velocities that were interrogated, it actually undergoes small frequency changes that can induce small errors in the measurement if they are not taken into account. It is also important to note that the increase in the frequency of the three central transmission maxima related to changes in the temperature, Figure 6b is not the same, so it must also be taken into account in the calculation of the properties of the analyte under test.

## 4. Conclusions

A PnC liquid sensor with a temperature compensation mechanism by means of differential measurements was studied.

The PnC structure has three resonant defect modes. These modes are due to the breaking of the structural periodicity or translation symmetry of the PnC by three defect layers. The three peaks are located within the phononic bandgap and are very sharp in shape, indicating strongly localized defect modes. The behaviour of the system is very consistent with the 1D Fabry–Pérot cavity behaviour. The introduction of three defect modes instead of one causes the modes to have energy transfer between them. This interaction between modes or mode coupling splits the single peak into three new resonances around 2 MHz. When the properties of one of the three defect layers are varied the symmetry of these three defect mode resonances is disrupted and the peaks displace themselves in frequency. The avoid crossing behaviour is due to the energy transfer between modes critically slowing down the displacement of the peak that comes closer to the central maxima located at 2 MHz.

The working principle of the differential PnC sensor is performing a frequency tracking of three transmission maxima located at the centre of the bandgap. The introduction of these defect modes allows following the changes in the speed of sound that occur in the liquids contained in the central cavity of the structure, showing the effectiveness of the use of the symmetry reduction technique for the design of sensors with phononic crystals. Only the central cavity was filled with analyte, maintaining the two adjacent cavities as a reference, which allowed to make the central transmission maxima sensitive only to changes in temperature but not to those of speed of sound in the analyte. The temperature compensation mechanism used enables the realization of differential measurements with a single signal path. This facilitates the use of this sensor in applications where temperature control is a problem, such as conducting point of care tests at home or in the field.

## Figures and Tables

**Table 1:** Material properties of the PnC sensors used.

Layer #	Thickness (mm) PnC Sensor	Thickness (mm) Control Sensor	Material	ρ (Kg/m^3^)	c (m/s)
1	-	-	PZT ^1^	7500	3333
2	0.187	0.187	Water	998	1483
3	0.715	0.715	Glass	2200	5720
4	0.187	0.374	Water	998	1483
5	0.715	0.715	Glass	2200	5720
6	0.374	0.374	Analyte/Water	998	1483
7	0.715	0.715	Glass	2200	5720
8	0.187	0.374	Water	998	1483
9	0.715	0.715	Glass	2200	5720
10	0.187	0.187	Water	998	1483
11	-	-	PZT ^1^	7500	3333

^1^ The layer thickness of the PZT layers is considered as semi-infinite for the simulation.
